# Ligation of injured subclavian vessels saved a young life: a case report

**DOI:** 10.11604/pamj.2022.42.117.32980

**Published:** 2022-06-14

**Authors:** Sharfuddin Chowdhury, Abdallah Alferdaus

**Affiliations:** 1 Department of Trauma, King Saud Medical City, Riyadh, Saudi Arabia,; 2 Department of Surgery, King Saud University, Riyadh, Saudi Arabia

**Keywords:** Stab wound, subclavian artery, mediastinum, upper extremity, case report

## Abstract

We describe a rare life-saving case of penetrating subclavian vessel injury that was managed successfully at King Saud Medical City, a major trauma center in Riyadh, Saudi Arabia. The patient was a healthy 21-year-old Saudi male who was presented initially at Al-Aflaj Hospital, 300 km away from King Saud Medical City, following a stab with a knife to the left side of his lower neck. He was transferred to King Saud Medical City for definitive surgical management after having temporary bleeding control at Al-Aflaj Hospital. The patient was successfully managed with a median sternotomy, left supraclavicular extension, and clavicular division. Hemostasis was achieved by ligating the injured subclavian vessels in a situation of extremes to save a life. His postoperative course was uneventful. He was discharged on the eleventh postoperative day with an intact neurovascular condition of the left upper limb.

## Introduction

Subclavian vessel injuries remain rare and highly lethal, despite advances in modern medicine [1,2]. In pre-hospital settings, mortality from penetrating traumas involving the subclavian vessels can reach 60% [3]. Operating room mortality rates range between 5% and 30% [3]. In 1919, during World War I, only 45 subclavian arteries (SCA) injuries among British casualties were reported. During World War II in 1946, the incidence among American soldiers was less than 1% of all vascular injuries. In 1950, only three patients with SCA injuries were reported in a study that included 304 major arterial injuries during the Korean conflict [4]. The management of SCA injuries is incredibly challenging due to their difficult anatomical surgical exposure. The arch of the aorta gives off three main branches: the innominate artery on the right, the left common carotid artery in the middle, and to the left posteriorly and distally the left SCA. The SCA is divided anatomically by the scalenus anterior (SA) muscle on both sides into three parts. The first part courses from the origin to the medial border of the SA. The second portion lies behind the SA muscle, while the third part courses from the lateral border of the SA up to the lateral border of the first rib [4]. The branches of the SCA are the vertebral, internal thoracic, thyrocervical trunk, costocervical, and dorsal scapular artery. The first three branches arise close to each other at the medial border of the SA. There is a network of collaterals connecting the branches of the SCA to the axillary artery (AA), bypassing any occlusion or damage in the SCA [4]. The anatomic location and the proximity of three neural structures, such as brachial plexus, vagus nerve, and phrenic nerve, make the surgical exposure of subclavian vessels more challenging for the operating surgeon. Moreover, the proximal third of the SCA is located within the thoracic cavity, hindering exposure. We describe a rare life-saving case of penetrating subclavian vessel injury that was managed successfully at King Saud Medical City (KSMC), a major trauma center in Riyadh, Saudi Arabia.

## Patient and observation

**Figure 1:**
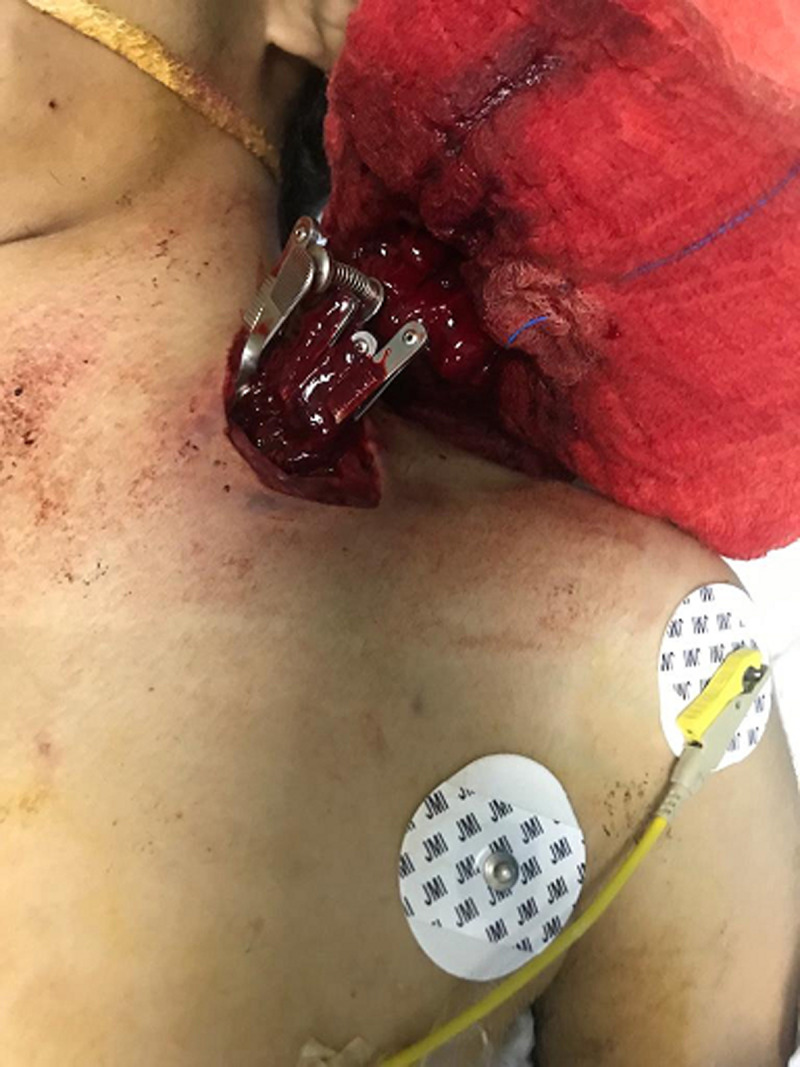
temporary bleeding control by multiple bulldogs (vascular clamps)

**Patient information:** a healthy 21-year-old Saudi man without relevant medical, family, and psychosocial history was stabbed with a knife by an unknown person on the left side of his lower neck around 7 pm on July 13, 2021, and presented to the nearest Al-Aflaj Hospital emergency department by a private vehicle. At Al-Aflaj Hospital, he was hemodynamically stable with active bleeding from the wound. The initial trauma resuscitation was done according to the Advanced trauma life support (ATLS) protocol. In the emergency department, they intubated him to control the airway, applied pressure dressings over the wound, and inserted a left intercostal tube (ICT) to drain the hemothorax. He was then shifted to the operating room (OR) immediately. The general surgeon extended the supraclavicular wound and applied multiple bulldogs (vascular clamps) to get temporary bleeding control (Figure 1). The patient was then transferred to KSMC trauma center as a life-saving case for definitive surgical management.

**Figure 2:**
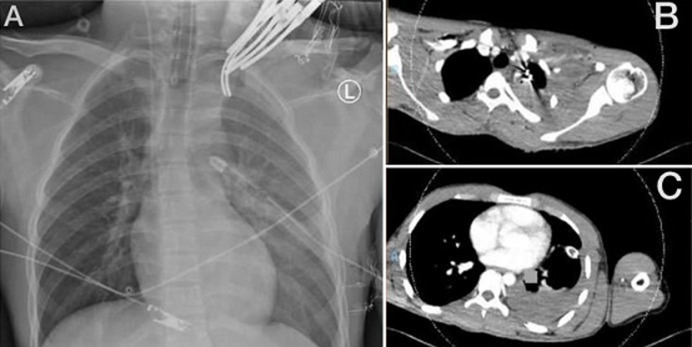
A) plain chest X-ray, anteroposterior view in the supine position at presentation; B) computed tomography scan of the lower neck/upper chest showing subclavian vessel injury; C) computed tomography scan of the chest showing left sided hemothorax

**Clinical findings:** the patient arrived at KSMC at 02:30 AM on July 14, 2021. The emergency department physician activated the trauma team, and the trauma team evaluated the patient immediately. On presentation, he was intubated and mechanically ventilated, with oxygen saturation of 100%. His heart rate was 92 beats per minute, and his blood pressure was 109/63 mmHg without isotropic support. He was sedated with reactive pupils. His left hand was cooler than his right hand, but well perfused (oxygen saturation 98-99% in all fingertips). There was no palpable pulse, but a Doppler signal was present in his left upper limb. Chest X-ray showed a left hemothorax (Figure 2 A), and the left ICT drained 700 ml of blood. The venous blood gases showed a pH of 7.35, bicarbonate of 21.7, PCO2 of 36.1, a base deficit of 3.4, lactate of 2.2, and hemoglobin of 7.6 gm/DL. The massive transfusion protocol was activated, and the OR personnel was alerted.

**Timeline of current episode:** the patient was stabbed in the left neck in the evening at 7 PM on 13 July 2021 and presented to KSMC in the early morning at 02:30 AM on 14 July 2021. After initial trauma assessment, resuscitation, and investigations, he was taken to the emergency OR on the same morning at 5 AM. He was discharged from the hospital on 25 July 2021 and had an outpatient clinic follow-up on 2 August 2021.

**Diagnostic assessment:** the patient was hemodynamically stable. He was shifted for computed tomography (CT) brain and CT angiography (CTA) for the chest, neck, and left upper limb. The CT brain was unremarkable. The neck and upper limb CTA showed a complete cutoff at the left SCA after its origin. A severe metallic beam-hardening artifact was causing difficult evaluation of the second part of the left SCA. There was also a hematoma surrounding the left axillary vein with a patent lumen (Figure 2 B). The left AA and brachial artery appeared patent. There was also a left moderate to a large amount of hemothorax (Figure 2 C). After returning from the CT, the patient became hypotensive and a transient responder to fluid resuscitation in the emergency department. The left intercostal drain output increased to 1.2 l in the next 2 hours.

**Diagnosis:** hemodynamically unstable penetrating left subclavian vessel injury.

**Figure 3:**
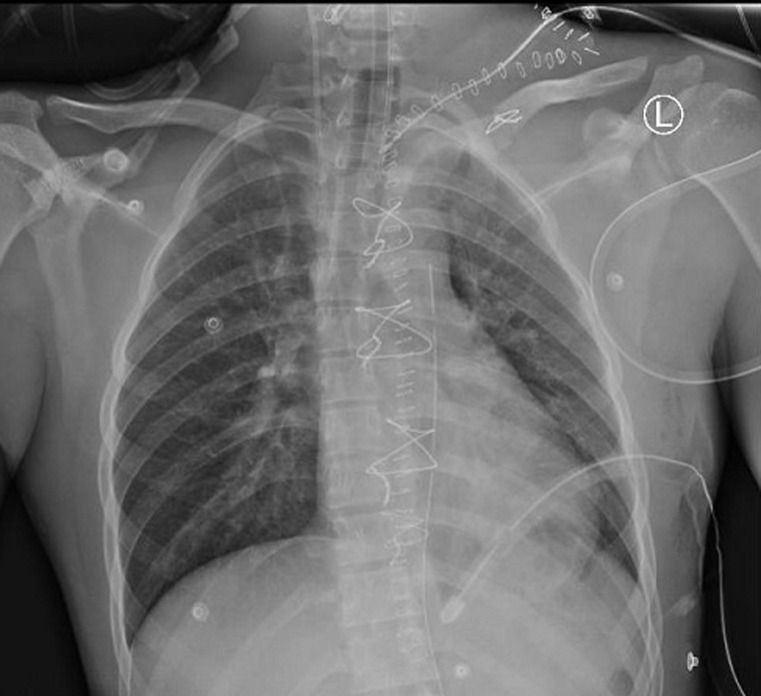
plain chest X-ray, anteroposterior view in the supine position on the first postoperative day

**Therapeutic interventions:** the patient was shifted immediately to the OR. Intraoperatively, a left supraclavicular incision with clavicular division was performed. Proximal control of the left SCA was difficult, and there was evidence of continuous blood oozing in the chest cavity. He became unstable on the OR table, requiring inotropic support to maintain hemodynamics despite ongoing blood transfusion. Immediate median sternotomy was performed (Figure 3). Two to three liters of blood were evacuated from the left chest cavity. The ligation of the injured subclavian vessels achieved hemostasis. The left upper limb showed good perfusion throughout the procedure, as evidenced by good pulse oximetry signals. His left upper limb was not significantly swollen, and there was no emergent sign of compartment syndrome. Therefore, we did not proceed with upper limb fasciotomy. The patient received seven units of packed red blood cells, four units of fresh frozen plasma, and four units of platelets during the procedure. Postoperatively, he was shifted to the intensive care unit for close monitoring.

**Figure 4:**
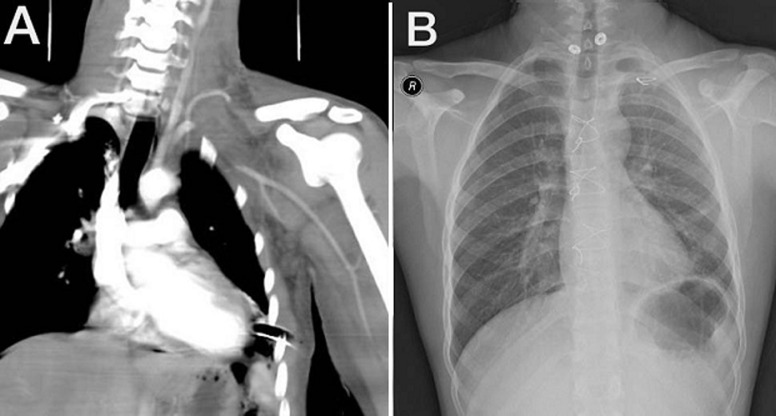
A) postoperative computed tomography angiogram of neck and upper limb; B) plain chest X-ray, posteroanterior view during the outpatient clinic visit

**Follow-up and outcome of interventions:** his postoperative course was uneventful. After gradual weaning from inotropes, he was extubated on the second postoperative day. He was moved to the general ward the following day. The ICT was removed on the ninth postoperative day. The limb was mildly swollen compared to the right limb, perfused throughout, and there was no neurological deficit. A follow-up CT angiography showed postsurgical intervention, with a finding of ligation of the proximal left SCA. Refilling of the SCA´s distal segment and its branches was noted down to the left upper limb (Figure 4 A). On the eleventh postoperative day, the patient was discharged with advice to use left upper limb compression stockings (class 2) for swelling. The outpatient clinic´s follow-up showed a well-perfused and neurologically intact left upper limb. His left upper limb swelling had reduced significantly and become almost normal. The chest radiograph was also unremarkable (Figure 4 B).

**Patient perspective:** the patient had no complaints when he was examined in the clinic following surgery, and the checkup was unremarkable. He conveyed his heartfelt appreciation for the surgical team.

**Informed consent:** written informed consent was obtained from the patient for publishing the article.

## Discussion

The rarity of subclavian vascular injuries in civilian trauma makes management extremely difficult for the attending physicians and surgeons. Demetriades *et al*., in a retrospective review, described balloon tamponade with a Foley catheter as an effective way for retro-clavicular injuries where direct pressure is often not possible. They also mentioned that the application of balloon tamponade might allow safe transfer of the victim to a trauma center or from the emergency room to the OR [1]. Later, Scriba *et al*. from Cape Town showed successful use of Foley catheter balloon tamponade in ninety-two (97%) such cases [5]. In our case, such intervention could immediately control bleeding in the emergency department of the referring hospital. However, we want to thank and acknowledge the surgeon at peripheral Al-Aflaj Hospital for his attempt to control bleeding in the OR by extending the wound, applying bulldogs, and facilitating safe transfer. Preoperative angiography helps in planning an operative approach for hemodynamically stable patients [6]. Patients with a hard vascular sign like active bleeding, large hematoma, or pulsatile hematoma should avoid visiting the CT suite for scanning [6]. In our case, we performed preoperative CT angiography as the patient was hemodynamically stable at presentation.

Right-side injuries can be treated with a median sternotomy and, if necessary, anterior cervical and/or right supraclavicular extension. Left-side injuries are best approached through a high anterolateral thoracotomy between the second and third intercostal spaces, which can be expanded with a median sternotomy and, if necessary, extended with a supraclavicular incision known as trapdoor access [3,6]. We started with an extension of the stab wound by the left supraclavicular incision, then divided the clavicle. This approach did not achieve proximal control of the left SCA, and there was evidence of active bleeding in the chest cavity. We immediately proceeded to median sternotomy for better exposure of the mediastinal vasculature. Scriba *et al*. support our approach for actively bleeding patients [5]. Endovascular repair of axillo-subclavian arterial injuries is a minimally invasive procedure that can be performed as an alternative to open repair. Branco *et al*., in a retrospective review of 153 axillo-subclavian injuries patients, concluded that endovascular repair was associated with a reduction in mortality and complication rates [7].

In our instance, endovascular repair was not an option, as the patient already had an operation at the referring hospital with multiple bulldogs placed in the wound. This would make guidewire cannulation and deployment of the stent during the endovascular intervention impossible. Moreover, in the middle of the night, the patient was a transient responder to fluid resuscitation and subsequently unstable. Such a situation eliminated the option for endovascular repair [3]. A strategy for damage control is usually used to treat a patient with a life-threatening injury that minimizes the duration of the procedure while remaining within physiological limits and improving the patient´s chances of survival [3]. We chose to ligate the vessels, as there was adequate perfusion by collaterals. An argument could be made to create a vascular shunt using a segment of tubular material with a compatible diameter, such as a nasogastric tube, that provides temporary perfusion to the limb/organ. However, due to the small neck at the second part of the SCA, insertion of a shunt would have been extremely difficult [3]. Moreover, we have seen covering of the left SCA during thoracic endovascular aortic repair appear safe, with no adverse effects on mental or physical health. Additionally, covering the left SCA does not increase the risk of upper extremity symptoms or impairment of normal activities over the long term [8]. This makes us feel comfortable with ligation of subclavian vessels in an extreme situation.

## Conclusion

Penetrating subclavian vessel injuries are rare and lethal. Foley catheter balloon tamponade is effective for neck and retro-clavicular injuries where direct pressure is often not possible. After initial resuscitation and temporary hemorrhage control, rapid transfer of a stable patient to an appropriate facility, preferably to a level 1 trauma center, is the cornerstone for a better outcome. In unstable patients, open repair is recommended without any delay. Ligation of the proximal SCA can be performed safely as a damage control measure to save the patient´s life and limb.
